# The Inclusive Behavioral Immune System

**DOI:** 10.3389/fpsyg.2019.01004

**Published:** 2019-05-03

**Authors:** Keren Shakhar

**Affiliations:** Department of Psychology, College of Management Academic Studies, Rishon LeTsiyon, Israel

**Keywords:** behavioral immune system, sickness behavior, social immunity, inclusive fitness, evolutionary psychology

## Abstract

Although living in social groups offers many advantages, it comes at a cost of increased transmissible disease. The *behavioral immune system* (BIS) is thought to have evolved as a first line of defense against such infections. It acts by minimizing the contact of yet uninfected hosts with potential pathogens. The BIS has been observed in a wide range of animals including insects, amphibians and mammals, but most research has focused on humans where the BIS is guided by complex cognitive and emotional processing. When researchers discuss the evolutionary origin of the BIS, they assess how it raises individual fitness. What would happen though if we shift our attention to the evolutionary unit of selection – the gene? Success would be measured as the change in the gene’s prevalence in the entire population, and additional behaviors would come to our attention – those that benefit relatives, i.e., behaviors that raise *inclusive fitness.* One widely-recognized example of the inclusive BIS is *social immunity*, which is prevalent among eusocial organisms such as bees and ants. Their colonies engage in a collaborative protective behavior such as grooming and the removal of infected members from the nest. Another example may be *sickness behavior*, which includes the behavioral, cognitive and emotional symptoms that accompany infection, such as fatigue, and loss of appetite and social interest. My colleague and I recently suggested that sickness behavior has evolved because it reduces the direct and indirect contact between an infected host and its healthy kin – improving inclusive fitness. These additional behaviors are not carried out by the healthy individuals, but rather by whole communities in the first case, and by already infected individuals in the second. Since they step beyond the classical definition of BIS, it may be useful to broaden the term to *the inclusive behavioral immune system*.

## Living in Groups Comes With Risks – Pathogen Exposure

Living in large social groups offers several advantages (Shultz et al., 2011). It reduces the risk of predation, simplifies the care for offspring and improves food foraging and food protection. Yet, it also has its drawback – it increases the risk for transmissible infections (McCallum et al., 2001). Pathogens can spread more easily among members of groups that exhibit social contact, share food and move around in the same territory (Cote and Poulin, 1995). Accordingly, both in animals and humans, social isolation is a common intervention that can contain infectious diseases by reducing transmission. Thus selective culling (Fèvre et al., 2006), quarantine (Fraser et al., 2004; Tognotti, 2013), school closures (Earn et al., 2012) and bans on travel and public gathering (Hatchett et al., 2007; Markel et al., 2007) have proven successful in containing epidemics.

Additional anecdotal support for the importance of social exclusion as a barrier for transmission comes from recent observations in bats, mice, and spiders. Recently, a fungal disease called White Nose Syndrome decimated several bat populations in North America (Blehert et al., 2009; Lorch et al., 2011). As some species approached the verge of extinction, several bat colonies escaped this fate by adopting a solitarily hibernating pattern in which individuals distanced themselves from their neighbors (Langwig et al., 2012). Relatedly, in mice and spiders, experiments have shown that “bold” individuals, which had more encounters with other conspecifics, had higher infection rates than “shy” individuals (Dizney and Dearing, 2013; Dearing et al., 2015; Keiser et al., 2016).

## Pathogens Exert a Strong Selective Pressure

Benefiting from modern medicine, complete with vaccinations, antibiotics, hygiene, and health care, we easily forget that until recently pathogens have been the leading cause of human mortality (Armelagos et al., 1996). Even our recent history is punctuated by repeated outbreaks of deadly epidemics (e.g., influenza, cholera, smallpox, polio, and HIV). Globally, life expectancy more than doubled during the past two centuries (Oeppen and Vaupel, 2002) and combatting infectious diseases was the leading cause for this increase.

Encounters with pathogens throughout our evolutionary history are heavily represented in our genome. Approximately 8% of the human genome is comprised of DNA originating from retroviruses (Griffiths, 2001), a subgroup of viruses that incorporate their genes into the DNA of host cells. Even this high percentage is a significant underestimation of our encounters with retroviruses since to be preserved in our DNA these retroviruses had to infect our germ cells and their integration needed to become fixed (Griffiths, 2001). Genetic variation among different geographically-distributed human populations also echoes the massive burden of infections as it is mostly attributed to pathogen exposure (Fumagalli et al., 2011). During the 100,000 years since humans migrated out of Africa and populations settled in different places, they encountered distinct pathogens which differently shaped their genome.

Clearly, pathogens exerted a tremendous selective pressure on humans and other animals. To avoid and survive infections, we had to evolve sophisticated mechanisms to resist them.

## Defense Mechanisms

There are several lines of defense that evolved to protect us against infections. These defense mechanisms interact with each other and act in a collaborative but also serial manner.

### Physical and Chemical Barriers

This so called “first line of defense” is comprised of surface barriers that block pathogen entrance into tissues. It includes the skin which serves as a physical barrier and mucous membranes that secrete buffering mucus and molecules that destroy many pathogens (e.g., gastric acid).

### The Physiological Immune System

If pathogens bypass the first line of defense and invade the body, than the physiological immune system comes into play. This is the most acknowledged and most studied pathogen defense mechanism. The proteins it utilizes are encoded by more than 7% of our genome (Kelley et al., 2005), reflecting the strong selective pressure from pathogens.

When a pathogen invades the body, the innate arm of the immune system acts immediately. If this response is insufficient, the adaptive immune system is called into play. It produces specialized cells and antibodies that specifically attack particular pathogens. Although effective in the long run, on the first time a pathogen is encountered there is a substantial delay until a specific immune response is fully deployed. Pathogens can exploit this delay to replicate rapidly and overwhelm their hosts.

On the longer run, pathogens develop strategies to evade the host’s immune response. In this ongoing arms race between pathogens and hosts the former enjoy a clear advantage: unlike humans who take decades to reproduce, most pathogens do so within hours or minutes so they rapidly evolve new strategies to evade or resist immune responses. The growing threat from bacterial resistance to antibiotics (Gootz, 2010) and the difficulty in developing highly efficient HIV and influenza vaccines (Carrat and Flahault, 2007; Barouch, 2008) are two examples for the rapid evolution of bacteria and viruses.

Overall, although the immune system is sophisticated and dynamic, its ability to combat pathogens is inherently limited. Perhaps a better way to oppose pathogens is simply to avoid contacting them.

### The Behavioral Immune System

In 2006, with this idea in mind, Schaller conceptualized a complementary defense system – the behavioral immune system (BIS) (Schaller, 2006). BIS is considered to be a motivational system that evolved to modify behavior and reduce contact with infectious agents. It is described as a coordinated suite of detection mechanisms, which allow individuals to identify potential sources of pathogens, and of emotional and cognitive mechanisms, which respond to those cues and guide behaviors that distance the individual from contagious sources (Schaller and Park, 2011).

Behavioral immune system likely has deep evolutionary roots. It is evident in a wide range of species: from insects through amphibians to mammals. Social lobsters and bullfrog tadpoles detect and avoid conspecifics infected with a lethal pathogen (Kiesecker et al., 1999; Behringer et al., 2006). In mice and rats several studies show that healthy individuals keep distance and reduce interactions with individuals whose immune system is activated (due to injections of the bacterial extract LPS) (Arakawa et al., 2011; Boillat et al., 2015). Mandrill monkeys avoid grooming infected conspecifics and refrain from the fecal matter (Poirotte et al., 2017). Thus, various groups of animals have developed behavioral strategies to detect and limit contact with pathogens and infectious individuals.

In humans, most support for the existence the BIS comes from extensive studies of the emotional and physical reaction commonly referred to as “disgust” (Curtis et al., 2004, 2011; Oaten et al., 2009; Tybur et al., 2013). We grimace, distance ourselves, and sometimes vomit in response to sights and smells of carcasses, decomposing food, bodily secretions, etc. Disgust often arises in response to potentially contagious elements and usually drives us away from them.

Additional support for the existence of the BIS in humans comes from studies showing that we can detect sick individuals through various cues. For example, the clothes of subjects whose immune system was activated (using LPS) can be sniffed out (Olsson et al., 2014; Regenbogen et al., 2017), and their gait and physical appearance are perceived as less healthy (Sundelin et al., 2015) and desirable (Regenbogen et al., 2017). Although not experimentally demonstrated, it is presumed that these signs lead to reduced contact with the potentially contagious individuals. One study has shown that priming with disease cues led individuals to describe themselves as less extrovert and less open to new experiences (Mortensen et al., 2010).

Since social psychologists were the leading researchers of the BIS, the field naturally centered on humans and its main interests became its effects on the social implications of erroneous detection (e.g., social categorization, prejudice and xenophobia, cross-cultural differences) (Park et al., 2003, 2007; Faulkner et al., 2004; Miller and Maner, 2012). Little experimental attention was given to how the BIS changes behavior toward infectious individuals and on the health outcomes of these dynamics.

## So what are we Missing?

Since the idea of the BIS as precautionary behavior seemed straightforward, not much consideration has been given to its precise definition. When Schaller first coined the term, he envisaged an anti-pathogenic defense system that detects potentially infectious organisms and objects and leads to a change in behavior (Schaller, 2006). Schaller was interested in how such a system that evolved to detect sick individuals affects social perception. He therefore focused on individual fitness – the emotions, cognitions, and behaviors that evolved to protect healthy individuals from contracting infections (Neuberg et al., 2011). If we recognize, as Schaller did, that an evolutionary process is driving the emergence of the BIS, than we must also acknowledge broader notions of natural selection. These have long placed the “unit of selection” at the level of gene, not the individual.

This shift in focus took form as Hamilton’s “inclusive fitness theory” over 50 years ago (Hamilton, 1964). This theory, also known as “kin selection” (Smith, 1964) was based on the realization that evolution occurs through the differential survival of competing genes. From the gene’s point of view, evolutionary success depends on leaving behind the maximum number of copies of itself in the population. Genes (more precisely, alleles – variants of a specific genes) whose phenotypic effects tend to increase their frequency will propagate through the population – regardless of which individual carries that gene. Thus, a gene may increase its evolutionary success by indirectly promoting the reproduction and survival of other individuals who also carry that gene and may do so even if they reduce the fitness of the individual originally displaying the phenotypic behavior. This idea is summarized by Hamilton’s rule which states that natural selection favors a gene that is costly to the individual carrying it whenever r*b > c, where r represents the genetic relatedness, b represents benefits, and c represents costs (Hamilton, 1964). Thus, if the reproductive success of relatives of individuals weighed by the probability of those relatives carrying the gene in question is larger than the cost to the survival and reproduction of that individual, this gene will be positively selected for.

But how could such a shift from the individual to the gene change our understanding of the BIS? It would broaden its definition to include behaviors that increase the fitness of genetically-related individuals who are likely to carry that gene. Thus, if an animal expresses a phenotype that reduces the transmission of a fatal disease to its kin, these kin (who are likely to also carry that gene and express this phenotype) would enjoy better chances to live and reproduce. Consequently, these genes would increase in frequency and this phenotype would be preserved even at the expense of the individual expressing it.

### Social/Collective Immunity

Social immunity is a term originally used to describe the collective defense mechanisms observed in eusocial insects (e.g., ants, bees, termites, and wasps) that result in avoidance, control, or elimination of infections (Cremer et al., 2007). The hallmark of these defense mechanisms is that single individuals cannot perform them efficiently. They require the collaboration of several individuals and are usually mounted for a collective benefit (Cremer et al., 2017). Although the term describes physiological, organizational as well as behavioral adaptations (Cremer et al., 2007), here I will only discuss their behavioral aspect.

Eusocial insects are clearly unique in their social interactions and tend to develop collaborative behaviors and altruism. Comprised of many closely-related sterile workers whose only path to reproductive success is to support their fertile queen and male kin, they act as a super-organisms (Wheeler, 1911; Boomsma and Gawne, 2018). The workers practice division of labor, collectively care for the brood, collect foraged food in communal stores and may sometimes defend the nest to their death (Shorter and Rueppell, 2011).

These colonies are very vulnerable for pathogen transmission as they live at high density, in constant physical contact with each other and even exchange food orally (Aubert and Richard, 2008). Their high genetic similarity also poses an additional risk as more individuals are susceptible to similar pathogens. Thus, eusocial insects are optimal candidates to develop social immunity. One interesting form of social immunity is social fever, which has been observed in honeybees (Starks et al., 2000). Fever has been acknowledged to improve survival following infections (Kluger et al., 1998). Its benefits have most convincingly been shown in exothermal animals such as lizards and fish (Kluger, 1991). Exposing them to sub-optimal environmental temperatures reduced their survival following infection. Fever is thought to improve resistance by depriving pathogens from the optimal temperature for growth as well as by improving the immune response (Kluger et al., 1998; Evans et al., 2015). In honeybees, workers decouple their wings and contract their flight muscles at high speed to elevate their temperature collectively speed and eliminate heat sensitive pathogens (Cremer et al., 2017). Thus, the behavior of healthy workers can eliminate pathogens affecting other members. It remains to be studied whether mammals also use social behavioral thermoregulation like huddling while infected.

In recent years the term social immunity has occasionally been broadened to mechanisms observed in non-eusocial animals. Allogrooming, in which individuals groom other conspecifics, is the most familiar form of behavioral social immunity. It has been observed in species ranging from insects, through birds, to a variety of mammals (primates, rodents, and ruminants). Although it serves an important function in establishing social networks and relationships, it clearly contributes to the hygiene of the animals by removing parasites. Although the behavior of the donor seems to contribute to the health of the recipient at the cost of the donor’s time and energy, allogrooming has been suggested to benefit the donor as well. In social ants for example, social contact including allogrooming of infected ants has been shown to immunize the naïve donor and increase survival upon later exposure to the same pathogen (Ugelvig and Cremer, 2007; Konrad et al., 2012). Thus, although this behavior has traditionally been interpreted in terms of kin selection in social insects, other forms of selections may take role in its evolvement. In vertebrates, reciprocal altruism is thought to play a larger role (Clutton-Brock, 2009), although kin selection may also have some function (Ju and Lee, 2016).

Aside from grooming, several studies of social insects have shown additional hygiene behaviors that reduce the spread of infections. These include removal, killing and burial infected nestmates (Cremer et al., 2017), sometimes of even before they become contagious (Pull et al., 2018).

In social immunity, healthy conspecifics engage in behaviors that reduce or eliminate the risk for infection in the colony. Although it has some costs for the healthy individual (e.g., it may increase its risk of being infected, consume time, or deplete energy), some forms of social immunity may provide direct benefit to the donor, making it more likely that social immunity is not limited to eusocial animals and can be found also in sub-social insects (Van Meyel et al., 2018). Whether or not vertebrate behaviors can also be interpreted using similar terms is still contentious.

### Could Sickness Behavior Be Understood as Part of the Behavioral Immune System?

When infected with a pathogen, many species display behavioral responses termed “sickness behavior” (SB) (Hart, 1988; Aubert, 1999; Dantzer and Kelley, 2007). SB includes depression, lethargy, hypersomnia, anorexia, reduced drinking, diminished libido, social withdrawal, and reduced grooming. Although some pathogens (e.g., rabies, toxoplasma) may manipulate behavior directly (Poulin, 2010), SB is in fact a well-orchestrated reaction produced by the host’s immune system (Dantzer et al., 2008; McCusker and Kelley, 2013). Infectious agents display a variety of pathogen-associated molecular patterns (PAMPs) that are recognized by receptors (e.g., Toll-like receptors) on various cells of the innate immune system (e.g., macrophages and dendritic cells). As a results these cells release proinflammatory cytokines (e.g., interleukin-1 and tumor necrosis factor alpha) that affect the brain through neuronal and humoral routes and induce SB (McCusker and Kelley, 2013). Even non-infectious agents (e.g., LPS, inactivated vaccines) that stimulate the immune system can induce SB. Blockade of the proinflammatory cytokines released by immune cells prevents SB, giving the ultimate support that SB is indeed induced by the host’s immune system (Bluth et al., 1992; Dantzer, 2006). The fact that SB is triggered by most infections, is orchestrated by the immune system, and persisted throughout evolution, suggests that it plays some important adaptive role in host defense (Hart, 1988).

We are so used to the manifestations of SB that we consider them the essence of being sick. In fact they are quite baffling as they carry significant adaptive costs to healthy animals (Moret and Schmid-Hempel, 2000; Hanssen et al., 2004). They can put the animal at higher risk of predation, of losing its territory and its social position, of dehydration and of starvation. In addition, these behaviors decrease parental care, and waste opportunities for reproductive success. To be preserved throughout evolution, these costs must be balanced by advantages.

My colleague and I (Shakhar and Shakhar, 2015) have recently proposed that SB has evolved because it reduces the risk of transmitting an infectious disease to offspring or other kin – a theory termed the Eyam Hypothesis after the English mining community that isolated itself to contain an outbreak of bubonic plague in 1666. Three-quarters of the villagers reportedly died, but the surrounding communities were saved (Massad et al., 2004).

Accordingly, self-imposed isolation characterizes most aspects of SB. For some symptoms it is obvious that they reduce the interactions of the infectious host with conspecifics. Such symptoms include depression, lethargy, hypersomnia, social withdrawal, and reduced grooming. Similarly, reduced libido limits courtship and mating behaviors. It is less clear how some symptoms, such as anorexia and reduced drinking, reduce contact and transmission. Presumably, when animals lose their appetite and thirst they share fewer meals with group members, do not contaminate the food or water supplies of the group, and defecate and urinate less, thus spreading less contagious pathogens to the environment.

Since SB often overlaps with the most infectious period of illnesses (Fraser et al., 2004; Carrat et al., 2008; Charleston et al., 2011), the reduced social interactions and contamination of the environment during this period likely reduce the transmission of pathogens. A study in mice has shown that 40% of mice injected with LPS reduced their social interactions with unchallenged mice (Lopes et al., 2016). Using a mathematical model that was developed based on these results the authors predicted that even if only 10% of mice had reduced their interaction, it would result in reduced transmission rate (Lopes et al., 2016). Although the change in behavior did not depend on genetic relatedness (Lopes et al., 2018), this species tends to live in close proximity to its kin (Rusu and Krackow, 2004) and thus social co-habituation may serve as a proxy for kinship.

Thus, if we redefine BIS according to the modern genetic perspective, it could incorporate SB. Individuals carrying genes for pronounced SB limit their social interactions when sick and protect their relatives (along with others) within the local group. SB could actually be a protective behavior that increases inclusive rather than individual fitness.

Accordingly, in eusocial insects, where genetic relatedness is very high and colonies are crowded, we can expect the behavior of infected individuals to be more dramatic. Indeed, social ants and bees infected with a pathogen or treated with (LPS) move less (Aubert and Richard, 2008), interact less with other ants (Bos et al., 2012), avoid contact with brood (Ugelvig and Cremer, 2007; Bos et al., 2012), spend more time outside their nest (Bos et al., 2012) and perhaps even sacrifice themselves (Rueppell et al., 2010). A recent study in ants demonstrated that after exposure to fungi spores, both exposed and non-exposed individuals adjusted their behavior to reduce the risk of contaminating their social network (Stroeymeyt et al., 2018). Clearly the case of super-organisms is unique as selection may occur at colony level (Wheeler, 1911; Boomsma and Gawne, 2018) but the extremity of such acts may provide support to the strong selective pressure pathogens have put on the behaviors of sick individuals in looser social networks.

Since the idea that SB may be part of the BIS and may have evolved to protect our kin from being infected is relatively new, only few studies that examine its premises exist. If this idea is true, we would expect SB to be stronger when pathogens are more virulent, when there is a greater chance of transmission either due to environmental characteristics (e.g., living in dense colonies) or pathogen’s characteristics (e.g., infectivity), and when the average genetic relatedness within group is higher than in the a. In addition, if SB is suppressed (e.g., through anti-inflammatory drugs), we would expect transmission rates to increase.

### Could Signaling Behavior by Infected Individuals Trigger BIS Responses by Healthy Recipients?

The BIS concept began with the notion that healthy people can detect contagious individuals. It is assumed that evolution equipped healthy individuals with the ability to detect cues of infection. But the concepts of social immunity and the Eyam hypothesis suggest a complementary possibility: that infected individuals actively emit “sickness signals” to warn their conspecifics and keep them away.

Findings from eusocial insects may support this concept. Termites that have contacted fungal spores vibrate to signal their group members that they have been infected. In response, other termites keep their distance from the infected area (Rosengaus et al., 1999). The health state of infected honeybee larvae and pupae can be smelled by the worker bees, leading to their weeding out from the hive, a phenomenon termed “hygiene behavior” (Wilson-Rich et al., 2009; Baracchi et al., 2012). Not only social insects, but mammals as well may signal their health status. Several studies in mice and rats show that LPS-treated individuals emit olfactory signals that drive other group members away and discourage interactions (Kavaliers et al., 2005). It is easy to accept that the detection of infected conspecifics has evolved as a protective mechanism against transmission. But, through kin selection, evolution may have favored not only individuals who can detect such cues but also individuals who display them. This is a subtle issue as infectious animals need to hide their status from predators but convey it to their kin. Experimentally testing this idea is difficult as it is hard to tease apart whether behavioral and sensory signs are perceived so only because of selection at the level of the non-infected individual (i.e., detection) or also at the level of the infectious individual (i.e., signaling).

## Conclusion

The BIS is a recently developed concept used to describe anti-pathogenic behaviors that evolved because they reduce the risk of infection. It has been assumed that this system evolved because it increases individual fitness. This paper proposes that individuals can also increase their inclusive fitness by protecting relatives (including offspring) from infection through kin selection. Thus, BIS can evolve due to its benefit not only to the individual fitness but also by indirect fitness of the individual by helping others.

Adopting this new outlook broadens the definition of the BIS to include additional behaviors such as social immunity and SB. Whether carried out by a healthy individual in the first case or by infected hosts in the second, these two kinds of behaviors are unique – they benefit others at the expense of the individual displaying them. Perhaps it is time to step beyond the idea of BIS and broaden the term to “inclusive behavioral immune system.”

## Author Contributions

The author confirms being the sole contributor of this work and has approved it for publication.

## Conflict of Interest Statement

The author declares that the research was conducted in the absence of any commercial or financial relationships that could be construed as a potential conflict of interest.
